# Three-Dimensional Manufacturing of Mandibular Total Edentulous Simulation Model for In Vitro Studies—Concept and Validation

**DOI:** 10.3390/polym17131820

**Published:** 2025-06-30

**Authors:** Joana Mendes, Maria Cristina Manzanares-Céspedes, José L. Esteves, João Fonseca, Lara Coelho, José Manuel Mendes

**Affiliations:** 1 UNIPRO—Oral Pathology and Rehabilitation Research Unit, University Institute of Health Sciences IUCS/(CESPU), 4585-116 Gandra, Portugal; joana.silva.mendes@iucs.cespu.pt (J.M.); mcmanzanares@ub.edu (M.C.M.-C.); lara.coelho@iucs.cespu.pt (L.C.); 2 Departament de Patologia i Terapèutica Experimental, Facultat de Medicina i Ciències de la Salut, Universitat de Barcelona (UB), 08007 Barcelona, Spain; 3 Human Anatomy and Embryology Unit, Faculty of Medicine and Health Sciences, University of Barcelona, 08007 Barcelona, Spain; 4 Department of Mechanical Engineering, Faculty of Engineering, University of Porto, 4200-465 Porto, Portugal; jesteves@fe.up.pt; 5 Department of Dental Sciences, University Institute of Health Sciences (IUCS), Cooperativa de Ensino Superior e Politécnico Universitário–CESPU, 4585-116 Gandra, Portugal; joao.fonseca@digital4all.com.pt

**Keywords:** additive manufacturing, edentulous simulation model, elastomer mucosa simulation, testing machine model, design

## Abstract

Background: Stereolithography is a rapid prototyping and 3D printing technique that creates solid three-dimensional models. An accurate and functional 3D model using stereolithography is invaluable in scientific research, particularly in studies involving edentulous patients. Additive manufacture and CAD systems help achieve accurate measurements and procedures and be easily replicated by lowering human error mistakes. The main objective of this study was to develop an in vitro simulation model with a reduced alveolar ridge with the same characteristics as mandibular edentulous patients using stereolithography. Methods: A mandibular model with a resorbed mandibular crest was scanned, and the STL model was aligned to the XYZ reference system. A reduction in the alveolar ridge corresponding to the mandibular mucosa of an edentulous patient was achieved. A negative model also derived from the original model was made to ensure the space for oral simulation material. A dimensional stability test was performed to validate the model. Results: The maximal mean displacement of the model was 0.015 mm, and the minimal mean displacement was 0.004 mm. The oral mucosa had a displacement of approximately 1.6 mm. Conclusions: An in vitro 3D simulation model of a complete edentulous patient mucosa was achieved.

## 1. Introduction

Digital dentistry has evolved exponentially, driven by the integration of CAD/CAM software, which is used for designing and manufacturing prototypes, finished products, and production processes [1].

Advancements in digital technology have significantly transformed clinical dentistry, and these changes have rapidly spread to other fields of science and research. Today, digital systems play a crucial role in the development of innovative approaches in oral rehabilitation research. Specifically, in dental material testing, digital technologies have contributed to the advancement of model and material fabrication through additive manufacturing. This technology has facilitated the standardization of testing methods, improving accuracy and reproducibility [2,3,4].

CAD/CAM systems have been helpful in the fabrication of prosthetic components, particularly in the production of crowns, complete dentures (CDs), and simple and complex surgical guides, as well as study and working models. These systems enable the seamless transition from digital design to the final manufactured product [5,6].

Unlike subtractive machining, commonly referred to as milling, additive manufacturing (AM)—better known as 3D printing (3DP)—has emerged as a transformative technology in dentistry. Integrated into CAM hardware, this process enhances manufacturing adaptability by producing prosthetic components layer by layer. The International Organization for Standardization (ISO/TC261) and the American Society for Testing and Materials (ASTM) define additive manufacturing as a “process of joining materials to create objects from 3D model data, typically layer upon layer” [7]. This method allows for greater flexibility in shaping components while ensuring precise topographic geometry. Among the various AM technologies available, stereolithography (SLA) is the most commonly used in dentistry [3,8,9,10,11,12,13].

There are numerous ways to conduct research in oral rehabilitation and prosthodontics; however, in vitro laboratory tests require models that characterize the patient condition. To reproduce and manufacture a model replicating the oral characteristics of a completely edentulous individual, several prototypes have been developed [14,15]. With the development of new technologies, particularly the advancement of digital technology and AM, the digital workflow has been enhanced [16].

SLA is a rapid prototyping and AM technique that creates solid, three-dimensional models. This AM process builds models layer by layer using data obtained from digital scans. It involves a resin-filled container, where the material solidifies upon exposure to ultraviolet (UV) light, creating an SLA prototype [17,18]. AM allows the production of complex prototypes with three-dimensional reproductions, which are indicated for manufacturing surgical guides and both diagnostic and surgical models [19]. Thus, several studies have highlighted the benefits of SLA, including its importance in those areas [19,20,21,22].

These SLA prototypes are invaluable in scientific research, particularly in studies involving edentulous patients. They are used in in vitro evaluations of the retention, stability, and adaptability of CDs in simulation models that replicate the alveolar ridges of patients with severe ridge resorption [12,23]. Numerous studies have assessed the retention of CDs, exploring both the use of endosseous implants and the application of dental adhesives to enhance denture stability [24,25].

Removable complete dentures (RCDs) are widely used in the treatment of edentulous patients. The stability and retention of these prostheses may depend on dental implants or simply adhesion to the oral mucosa. Despite advancements in dental rehabilitation, conventional CDs remain the first choice, particularly among elderly patients, due to economic factors [26,27]. The characteristics of CDs differ between the maxilla and the mandible. Previous studies have reported that maxillary RCDs are generally better tolerated by patients than mandibular ones. The primary source of dissatisfaction with mandibular RCDs is the lack of retention and stability [28,29].

A dental model is a replica of a patient’s oral structures, including the teeth and adjacent tissues. In completely edentulous patients, an accurate representation of soft tissues is crucial for conducting scientific research. However, plaster models have inherent limitations, such as susceptibility to deterioration due to the material properties [30]. In prosthodontics, AM models play a significant role, as they can be easily modified and adjusted as needed. These features are essential for their application in scientific research methodologies [31,32]. A simulation prototype of a completely edentulous patient must be able to allow an evaluation of the mechanical factors that influence the retention of dentures. An in vitro study must be carried out to reproduce an environment that is similar to the real one, which, in this case, is the oral cavity, where mechanical factors influence their retention [33]. Combining the technological world and digital systems, we intend to develop a 3D model using the AM method to reproduce the physical properties of oral tissues [34].

The main objective of this study was to develop an in vitro simulation model with a reduced alveolar ridge with the same characteristics as mandibular edentulous patients using SLA. The null hypothesis was that the in vitro simulation model has no stability to perform mechanical tests, presenting a displacement superior to 0.05 mm.

## 2. Materials and Methods

### 2.1. Study Design

In the present study, a simulation model of a completely edentulous patient with severe ridge resorption was developed. The oral mucosa was also simulated in this model. All the test materials and methods are described in detail below. The 3D prints of the simulation model were obtained under identical printing and processing conditions, including printing, washing, and post-curing parameters.

### 2.2. Materials

All the materials used were selected based on their importance and usefulness in dental medicine, as well as their stability under normal conditions of use and storage. All materials and chemicals were used as received without additional purification, unless otherwise indicated by the manufacturers.

#### 2.2.1. Development of the 3D Model Using Stereolithography

**Figure 1:**
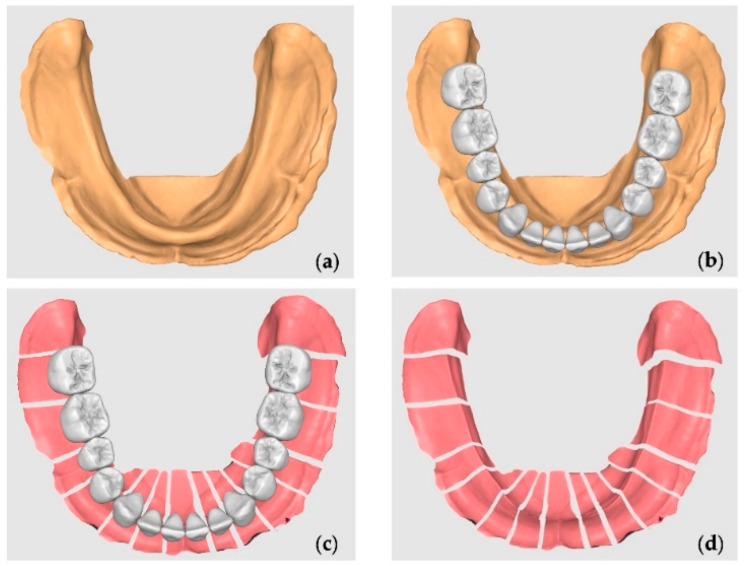
Mucosa reduction: (**a**) the STL model; (**b**) the standard tooth arrangement for a complete denture; (**c**) the section corresponding to the mid-distal space occupied by each tooth; (**d**) the custom negative offset without teeth.

**Figure 2:**
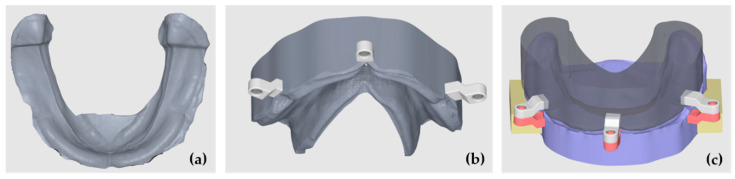
Oral mucosa thickness creation: (**a**) the reduction in the bone crest; (**b**) a negative model to ensure space for the artificial mucosa; (**c**) digital cobblestones and attachments design.

A mandibular plaster model with a resorbed mandibular crest, obtained from a Plaster Model Mold (Edentulous Jaw) by Nissin^®^ (Dental Products Inc., Minami-ku, Kyoto, Japan), was used as the basis for the AM process to generate the 3D model. The following sequence of steps was performed to create a digital model simulating the mucosal thickness over the resorbed bone:

1. 3D Scanning: The plaster model was scanned using a DS-EX Pro desktop scanner (Shining 3D^®^, Curitiba, Brazil), and the digital file was exported in STL format.
2. Initial CAD Preparation: The STL file was imported into Meshmixer^®^ (Autodesk^®^ Inc.), aligned with the XYZ coordinate system, and oriented parallel to the Y-plane to standardize positioning for modeling and comparison (Figure 1a).
3. Tooth Library Import and Positioning: A generic STL tooth library was imported containing teeth 47 to 37. The teeth were scaled proportionally and positioned over the crest to represent a standard full-arch tooth setup for a mandibular complete denture. The third molars were ignored and were not included in the section (Figure 1b).
4. Digital Model Duplication and Sectioning: The aligned STL model was duplicated. One copy was digitally sectioned in the mesio-distal direction at each edentulous tooth site, corresponding to the tooth axes (e.g., between 47 and 46, 46 and 45, etc.) (Figure 1c).
5. The Calculation and Application of Negative Offsets: In each digital section, a negative offset (i.e., inward shrinkage) was applied to simulate the mucosal layer thickness. The offset values were calculated based on the mean soft tissue thicknesses reported by Dong Jian et al. [27] for each specific site of the mandibular crest (e.g., ~2.0 mm anteriorly, ~3.0 mm posteriorly), as detailed in Table 1. This step effectively “reduces” the bone crest in each region by the average mucosal thickness, generating an estimated virtual mucosal profile (Figure 1d).
6. Model Recompositing: The offset-modified sections were digitally stitched together to reconstruct a full mandibular arch model with a virtual soft tissue layer removed, thus simulating the underlying bony ridge (Figure 2a).
7. Digital Tray Inversion: To generate a negative impression tray of the mucosa, the original (pre-offset) STL model was inverted using Boolean subtraction, resulting in an internal geometry that replicates the gingival surface of the mucosa (Figure 2b).
8. Finalization for Printing: The resulting models (reduced bone crest and negative mucosa tray) were made watertight, thickened, and prepared for resin printing. The wall thicknesses were adjusted to ≥2 mm to ensure structural integrity during additive manufacturing.

As the final result is a 3D simulation model for research testing, parallel cobblestones were added bilaterally to the model. These cobblestones were positioned parallel to each other, allowing the model to be easily placed and properly adapted on a transfer table in a fatigue-testing machine.

In order to guarantee the same material thickness between the final model with the simulation of the reduced bone crest and the digital mold, a generic male–female attachment in STL was imported, created digitally in the same software. The attachment was digitally duplicated and positioned so that the males were in the bone crest simulation model and the females were in the digital tray, bilaterally. The introduced space limiters ensured the uniform thickness of the mucosa simulation material throughout the area corresponding to the model’s mucosa. The digital cobblestones and attachments were joined to the model and the digital tray using a Boolean union operation (Figure 2c).

The digital bone model and the digital impression tray were exported in binary STL format to a folder, and, subsequently, the STL models were imported into 3D slicing software (Preform, Formlabs^®^ Inc., Berlin, Germany).

The 3D objects were positioned at a 30° angle relative to the base of the printing platform, as represented in the software. Printer media were created using the software’s proprietary algorithm. The objects were automatically sliced and exported to a 3D printer.

The 3D objects were printed in a white resin (White resin V4, Formlabs^®^ Inc., Berlin, Germany) on an SLA printer (Formlabs 3, Formlabs^®^ Inc., Berlin, Germany). The SLA material was chosen for its dimensional accuracy, mechanical rigidity (Young’s modulus ~2700 MPa), and ability to reproduce fine anatomical details. Its matte white color provides a high contrast for visual inspection and surgical simulation. This resin has been previously validated in medical applications requiring accurate anatomical modeling and is compatible with clinical digital workflows [36,37,38,39].

The printed models were immersed in an isopropyl alcohol solution for 18 min using FormWash (Formlabs^®^ Inc., Berlin, Germany) and then cured for 15 min in a UV unit (FormCure, Formlabs^®^ Inc., Berlin, Germany).

#### 2.2.2. Simulation Mucosa Elaboration

The obtained 3D upper and lower models were created in different ways. An insulating aqueous solution (Aislar, Kulzer^®^ Iberia S.A., Madrid, Spain) based on alginate, free from formaldehyde, was applied on the upper model, with the function of isolating surfaces, suitable for CAD-CAM models. This product produces a thin insulating film that can be easily removed manually when desired, and it aims to prevent elastomer adhesion and allow for the easy disinsertion of the upper model.

The simulation mucosa material used was the vinyl polysiloxane Ufi Gel^®^ SC (VOCO^®^ GmbH, Cuxhaven, Germany). The selection was made in accordance with the previous study performed by Mendes et al. [40].

In the lower model, the area corresponding to the oral mucosa was degreased with a compress moistened with 90% alcohol and left to air dry for 1 min. Then, with a brush, the adhesive (Ufi Gel SC, VOCO^®^ GmbH, Cuxhaven, Germany) was placed in the area corresponding to the mucosa in order to facilitate the adhesion of the material. In the remaining area of the mandibular 3D model, the same insulator for the upper model was applied.

**Figure 3:**
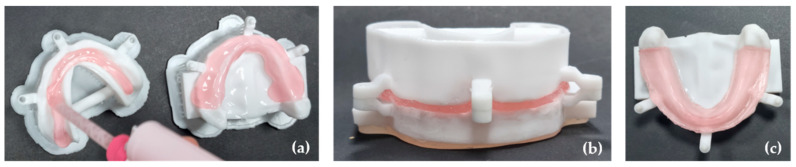
Oral mucosa adaptation: (**a**) the 3D mandibular and upper model filled with Ufi Gel^®^ SC; (**b**) the upper model placed over the mandibular model with the attachments joined to ensure the gingival thickness; (**c**) the final 3D simulation model.

Both the 3D mandibular model with a reduction to simulate oral mucosa tissues and the upper model were filled with the vinyl polysiloxane Ufi Gel^®^ SC (VOCO^®^ GmbH, Cuxhaven, Germany) (Figure 3a). The upper model, corresponding to the digital tray, was placed over the mandibular model and closed until the male and female attachments joined. This ensured the standardization of the material according to the previously described average gingival thicknesses, and the excess material was removed (Figure 3b).

The polymerization of the material was carried out by placing the closed models in a pressure cooker for 15 min at a temperature of 45 °C. Afterwards, the models were left to cool down at room temperature, and the negative model was removed. Finally, the peripheral sealing of the elastomer was performed, which corresponded to the simulated mucosa, using Ufi Gel SC glaze (VOCO^®^ GmbH, Cuxhaven, Germany). To harden the model, its base was also filled with the resin Orthocryl^®^ (DENTAURUM GmbH & Co., Ispringen, Germany).

The described procedures were performed three times. As a result, three 3D simulation models were obtained with the previously described average gingival thicknesses for in vitro studies (Figure 3c).

#### 2.2.3. Validation of 3D Model Stability

A study was carried out on the dimension stability of the three 3D simulation models when subjected to chewing forces of values between 0 and 250 N, with the vertical displacement being measured at three different points on the model base.

The three attachments placed in the model were used to ensure the gingival thickness once they were attached to the digital bone model without contact with the oral mucosa simulation material.

Thereafter, 250 N was selected as the maximum value for conducting this test, using the study by ELsyad et al. [41]. In this study, the bite force was measured in patients with three different rehabilitations or prostheses [41] The mean value between the maximum force of the rehabilitations and the larger/smaller values was calculated. The mean value was 226 N, but we used 250 N in order to ensure the stability of the model base, even with values above the average.

**Figure 4:**
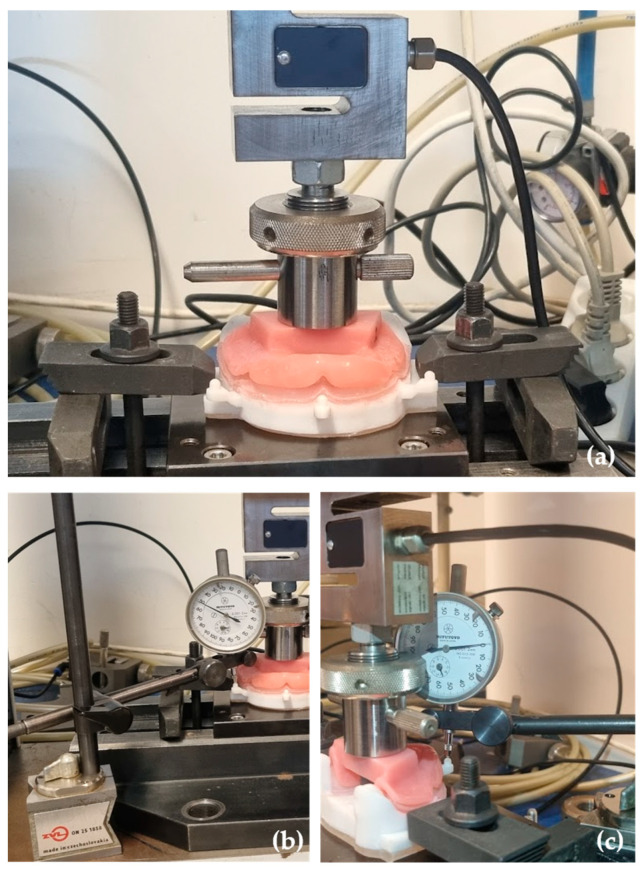
Model stability test: (**a**) the model and the prosthetic base adaptation in the Tira test 2705 machine; (**b**) the Mitutoyo dial indicator placed in the lateral attachment; (**c**) the Mitutoyo dial indicator placed in the central attachment.

A universal testing machine, Tira test 2705 (TIRA GMbh, Schalkau, Germany), with a 5 kN load cell, at a constant speed of 10 mm/min was used. The vertical displacement test was conducted with a prosthetic base, adapted to the 3D simulation model, ensuring a uniform load distribution on the simulated mucosa (Figure 4a). For that, the 3D simulation model was fixed to the machine table using the cobblestones, and the prosthetic base was attached with a screw at the loading cell.

Mitutoyo dial indicators (Mitutoyo MFG. Co., Ltd., Tokyo, Japan)—measuring equipment with an accuracy of 0.001 mm—were used for each measurement point. The Mitutoyo dial indicators were fixed to the testing machine by magnetic supports, and the measurements were taken in the left, right, and center attachment of the model (Figure 4b,c).

#### 2.2.4. Sample Size

The sample size was calculated with G*Power 3.1.9.4 [42], considering one sample *t*-test and expecting a high effect size (Cohen’s d = 0.9) with 5% significance and 80% power. Concerning these conditions, the minimum sample size was set at 9. Hence, each of the three models were tested three times, completing a total of 9 trials.

#### 2.2.5. Statistical Analysis

The data was analyzed with R, version 4.5.1 [43]. Normality was tested with the Shapiro–Wilk test. Descriptive statistics were presented as means (M) and standard deviations (SD) for continuous variables and as frequencies (*n*) and percentages (%) for categorical variables.

Test comparisons of each attachment type were performed with RM ANOVAs. The effect size was calculated with eta-squared (η^2^), considering the thresholds of 0.01 (low), 0.06 (moderate), and 0.14 (high), following Cohen’s guidelines [44].

Unilateral *t*-tests were conducted to compare the thresholds of stability difference for the center, left, and right attachments. The choice for unilateral tests was due to the need to compare the observed mean results to each threshold (≤0.005, ≤0.010, ≤0.015, and ≤0.020), representing the hypothesis H0: μ ≤ threshold. Additionally, a bilateral test was run to compare the observed mean results with a hypothetical difference of 0.

Significance was deemed as *p* < 0.05.

## 3. Results

Table 2 shows RM ANOVAs for left, right, and center attachments across a sequence of tests.

The results from the repeated measures ANOVA showed that the values across the three tests were consistently very close to one another for all the attachment locations, with only minimal variations. For the center attachment, the mean values decreased slightly from 0.0147 (SD = 0.0015) in Test 1 to 0.0140 (SD = 0.0010) in Test 2 and 0.0133 (SD = 0.0012) in Test 3. Although this subtle decline resulted in a relatively large effect size (partial η^2^ = 0.89) and an F-statistic of F(2, 4) = 16.00, the *p*-value remained marginal at 0.057, suggesting that the differences, while consistent, were still small in absolute terms.

For the left attachment, the values remained particularly stable across the tests: 0.0063 (SD = 0.0015) in Test 1, 0.0060 (SD = 0.0010) in Test 2, and 0.0067 (SD = 0.0006) in Test 3. These nearly identical values produced an F(2, 4) = 0.25, a non-significant *p*-value of 0.667, and a very small effect size (partial η^2^ = 0.11), indicating no meaningful variation over time.

Similarly, the right attachment also showed only minor differences, with mean values of 0.0050 (SD = 0.0010) in both Test 1 and Test 2 and a slight reduction to 0.0043 (SD = 0.0012) in Test 3. Despite a relatively large effect size (partial η^2^ = 0.67) and an F-statistic of F(2, 4) = 4.00, the *p*-value was 0.184, reinforcing that the differences observed were not statistically significant and the overall values remained closely aligned across the trials.

**Figure 5:**
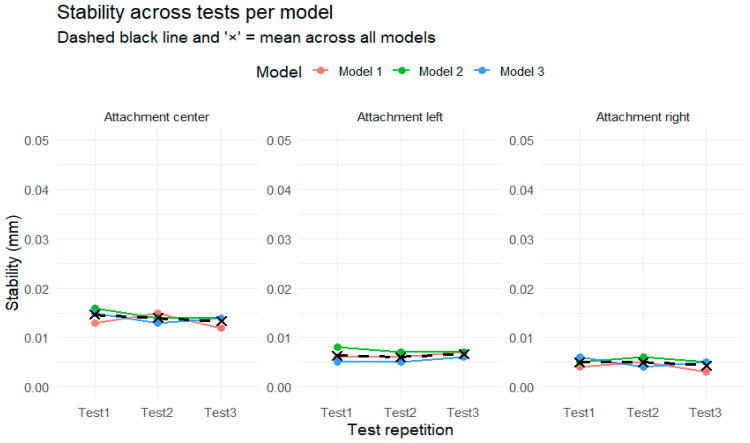
Stability across tests per model in each type of attachment.

Figure 5 illustrates the stability values (mm) across three repeated tests for each of the three models, separately for the center, left, and right attachments. Across all the conditions, the data show consistency between test repetitions, with only very minor variations between the models or over time.

In the center attachment, the stability values for all three models remained closely aligned and showed a slight decreasing trend from Test 1 to Test 3. However, the changes were minimal, and the individual model trajectories almost overlapped throughout. The overall mean stability, indicated by the dashed black line and the black “×” markers, remained nearly constant, reinforcing the observation that performance was highly stable across repetitions.

For the left attachment, the three models also demonstrated very similar values across the three test occasions, with the variations contained within a very narrow range. The plotted lines for each model followed almost parallel paths, and the overall mean remained flat.

The right attachment followed the same pattern of close agreement between models and near-identical values across tests. A minor decrease in values was observed in Test 3, but the differences were small and unlikely to be practically meaningful. The consistency of the dashed black line and the tightly clustered “×” symbols across all the tests highlights the overall stability of the attachment.

**Figure 6:**
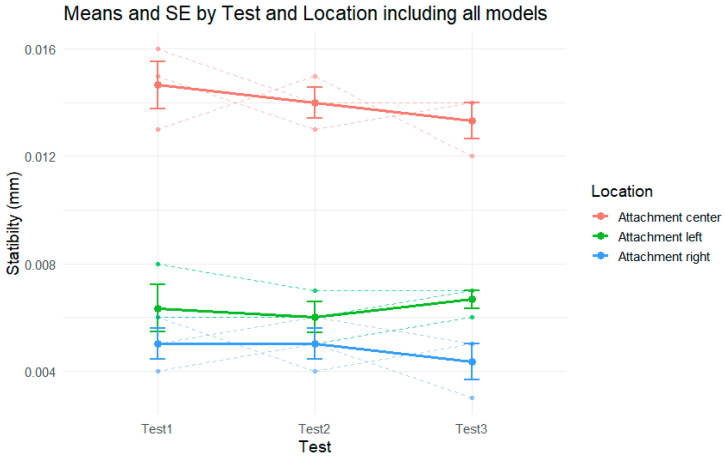
Stability by test in each attachment, including all models.

Figure 6 presents the mean stability (mm) and standard errors across three repeated tests, separately for each attachment location (center, left, and right), combining all the models. Overall, the results demonstrate a consistent pattern of very similar stability values across the test repetitions for each attachment, with only minor changes that fall within the range of the standard error.

For the center attachment (in red), the stability values show a slight decreasing trend across the three tests, from approximately 0.0147 mm in Test 1 to 0.0133 mm in Test 3. Although the means indicate a subtle drop in stability, the error bars and overlapping ranges suggest that the variation was small and consistent across the models.

The left attachment (green) presents nearly flat stability values throughout the tests. The mean values remained close to 0.006 mm across all the repetitions, with slight fluctuations that did not follow a clear directional trend. The standard errors remained relatively stable and small, indicating low variability and high consistency in performance.

For the right attachment (blue), a very slight decline in stability is observed, from around 0.0050 mm in Test 1 and Test 2 to approximately 0.0043 mm in Test 3. Similar to the other locations, the differences are minimal, and the error bars show overlapping values across tests.

**Figure 7:**
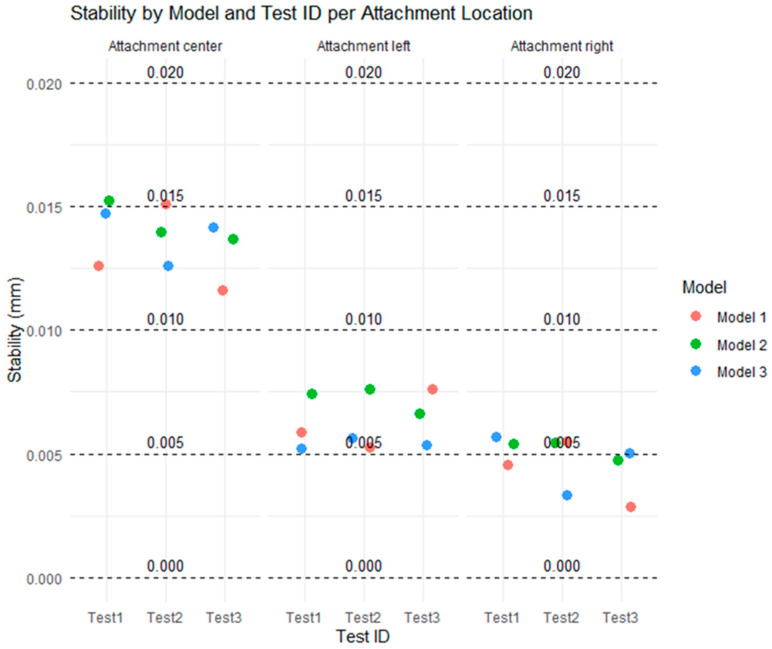
Distribution of all tests for types of attachment at all considered thresholds.

Figure 7 shows the distribution of stability (mm) for each combination of test (Test 1, Test 2, Test 3), model type (Models 1, 2, and 3), and attachment location (center, left, and right), in relation to five reference thresholds (0, 0.005, 0.010, 0.015 mm, and 0.020). Each point represents an individual stability measurement, allowing for the visualization of the spread of values.

At the center attachment, most values are concentrated between the 0.010 and 0.015 mm thresholds, indicating relatively higher instability in this location. All three models show similar values with small variations across the tests, and none exceed the 0.020 mm threshold.

At the left and right attachments, most of the values fall below 0.010 mm, with several points clustered at or below 0.005 mm. These results indicate that the lateral attachments exhibited lower instability levels compared to the center attachment. The differences between models are minimal, with Model 3 being slightly more consistent on the left and Model 2 showing slightly higher values on the right, although all the models remain within acceptable limits.

Overall, the figure shows that stability was higher in the lateral attachments, with lower dispersion and smaller values. The distribution of points relative to the thresholds highlights that the stability values remained within a safe range (below 0.020 mm), with the 0.005 mm and 0.010 mm thresholds particularly useful in distinguishing small variations across the models and tests.

**Figure 8:**
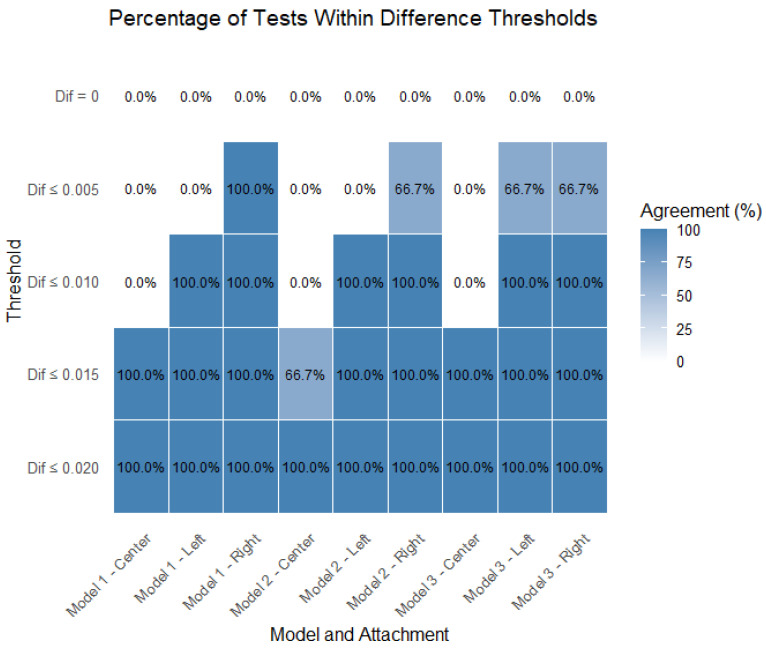
Percentage of tests within thresholds, separated by model and attachment.

Figure 8 displays the percentage of tests that remained within each of the defined stability thresholds (0 mm, 0.005 mm, 0.010 mm, 0.015 mm, and 0.020 mm), separated by model (Model 1, Model 2, and Model 3) and attachment location (center, left, and right).

For the center attachment, all the models show lower percentages of tests within the stricter thresholds (0.005 mm and 0.010 mm), especially for Model 1 and Model 2. However, nearly all the tests fall within the 0.015 mm threshold, indicating that while central attachments tend to have higher instability, they still meet the broader acceptability criteria.

For the left attachment, there is a marked improvement in performance across all the models. A higher percentage of tests falls within the 0.005 mm and 0.010 mm thresholds, particularly for Model 3, which shows the best performance in this location. Model 1 shows slightly lower percentages but still maintains good results.

For the right attachment, the results are comparable to those of the left attachment, with all the models achieving high percentages within the 0.010 mm and 0.015 mm thresholds. Model 3 again tends to outperform the others, especially in the strictest threshold (0.005 mm), suggesting better consistency in minimizing instability.

**Figure 9:**
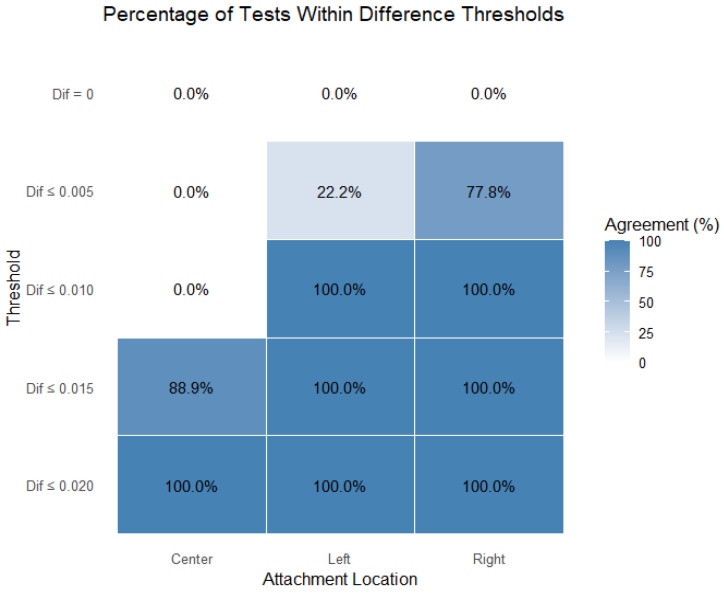
Percentage of tests within thresholds, separated by attachment.

Figure 9 presents the percentage of tests within the thresholds, separated by model and attachment; therefore, it includes all the models (*n* = 9 per attachment). The results confirm that the right attachment was the most consistent when falling closer to more-strict thresholds (closer to zero). The left attachment closely followed. The center attachment was the stability parameter that departed most from the lower thresholds, though it was also considered to be close to zero (88.5% of test results within the ≤0.015 threshold).

Table 3 summarizes the results of negative one-sided *t*-tests, comparing the mean stability values for each attachment location (center, left, right) against increasingly lenient difference thresholds (0.000, 0.005, 0.010, 0.015, and 0.020 mm), with *n* = 9 tests per location.

For the center attachment, the mean stability (M = 0.0140 mm, SD = 0.0012) was significantly greater than all the thresholds up to 0.010 mm (*p* < 0.001), but not significantly different from 0.015 mm or 0.020 mm. This indicates that the stability at the center exceeds the strictest thresholds but aligns with more lenient ones.

For the left attachment (M = 0.0063 mm, SD = 0.001), the stability was significantly greater than 0.000 and 0.005 mm (*p* < 0.001 and *p* = 0.002, respectively), but not significantly greater than 0.010 mm or the higher thresholds, suggesting that its stability falls comfortably within the moderate and lenient thresholds.

The right attachment (M = 0.0048 mm, SD = 0.00098) also had a mean stability significantly greater than 0 mm (*p* < 0.001) but not significantly greater than 0.005 mm (*p* = 0.256), nor any higher thresholds. This implies high stability at the right attachment, well within the standard limits.

## 4. Discussion

This study aimed to develop a model for in vitro laboratory tests that reproduced an edentulous mandible with alveolar ridge resorption with the physical properties of oral tissues by SLA. Dimensional stability tests were performed in order to ensure that there was no movement of the digital bone model during mechanical tests. The maximum value of displacement was set at 0.05 mm, because it will not affect mucosal displacement calculations. All the tests were performed by a single operator. The results show that the mean displacement value of the 3D simulation model was lower; therefore, the null hypothesis was rejected.

Several authors have described the development of simulation models of complete edentulous patients. However, these models were created using traditional techniques and manufactured using analog methods, without the rigor of digital technology and AM, increasing the risk of errors in their production. Johnson et al. [45] carried out a study in which they produced a simulation model of an edentulous mandibular in plaster. Additionally, Mendes et al. [46] developed an in vitro mandibular simulation model, which reproduced the environment and physical properties of oral tissues. Traditional techniques, like plaster, may also be affected by the presence of internal bubbles due to technical error, deterioration, and material properties [30].

Digital models, carried out in manufacturing processes using SLA, which is a technology used in AM, improve predictability and simplify the manufacturing procedures compared to those of traditional methods [47,48]. It is also known that CAD programs can produce accurate and clinical acceptable designs, even when using different programs [49]. Mistry et al. [47], in their study, showed that SLA is a viable technique capable of producing accurate and cost-effective study models with slight tolerance in variation accuracy. Another study that evaluated the dimensional stability of several 3DP methods shows that both the Polyjet and SLA techniques produced models that expanded buccolingually and anterioposteriorly; however, smaller differences were found vertically, especially using SLA Ref. [50]. For those reasons SLA was used in our study.

The 3D models’ adaptation to the mechanical testing machines is a primary issue, especially when using traditional methods [45,46]. This step is very important to ensure the reproducibility of the fatigue tests and to ensure that the model is accurately positioned during the test performance. In order to improve this condition, in our study, cobblestones were also included in the CAD procedure so that it could be easily adapted to the transfer table of a universal testing machine.

The model was created with the main purpose of reproducing the oral mucosa of a completely edentulous mandibular patient. Therefore, it was necessary to maintain the space corresponding to the mucosa of a completely edentulous patient with a reabsorbed alveolar crest. To ensure the various thicknesses of the mucosa in various locations of the jaw, we created three male–female attachments. According to Revilla-León et al. [10], custom trays manufactured using additive manufacturing technology with a CAD allow one to create a homogeneous space for the molding material. Therefore, in our study and following this principle, we inverted the initial model in order to convert it into a digital mold. The interior of this digital impression tray represented the surface of the reduced alveolar ridge with the same characteristics as mandibular edentulous patients.

The measurement of the gingival thickness was obtained through an average calculated by the results described by Dong et al. [35]. This is a limitation of this study, since measurements performed using the CBCT of different lingual and buccal areas were not used, but, instead, a mean value for each location of each tooth was used.

The space created was filled with the Ufi Gel^®^ SC (VOCO^®^ GmbH, Cuxhaven, Germany) elastomer, characterizing the alveolar mucosa. This elastomer was selected in another study conducted by Mendes et al. [38], which indicated that Ufi Gel^®^ SC and Molloplast B could be used as oral-mucosa-simulating materials. Although Molloplast^®^ B was found to be the most similar material, as stated by the authors, it is very difficult to work with. In the current study, Molloplast^®^ B was the first elastomer that the authors tried to use to simulate the oral mucosa; however, its adhesiveness and the fact that it is a heat-cured material made it impossible to use to obtain an accurate 3D simulation model. So, as Ufi Gel^®^ SC had good results, it was used, making it possible to conduct the model [40].

The dimensional stability test primarily showed that the 3D simulation model had a bigger displacement of the center attachment; however, the right and left attachments were more stable. This may be because the cobblestones that fixed the model onto the testing machine were located near that location, allowing the 3D simulation model to oscillate more in locations where there were no stabilizing attachments.

A study regarding 3D printed dental models referred to the fact that external forces are the principal cause of model damage, which suggests that the mechanical stress that is applied to the model has an important role in its quality [51]. In our study, the simulation model was impressed into a resin that allows us to conduct mechanical tests, supporting the appliance up to 250N, a value greater than that required in tests with complete edentulous rehabilitations [41].

Alshaibani et al. [52] concluded, in their study, that high-temperature storage leads to the lower stability of the simulation model, advising a stable room temperature. All the samples and models were stored at room temperature, and kept at room temperature during the dimensional stability test, in order to maintain model stability.

The minimal height of the oral mucosa in the current model was 1.7 mm; thus, the total displacement was 1,6 mm, being in accordance with Żmudzki et al. [53]. In their study, they stated that the extent of the mucosal deformation caused by a prosthetic base should not exceed the thickness of the mucosal membrane. Compagnoni et al. [54] also conducted a study and found that the maximal displacement value of the denture was 1.7 mm, with a mean value of 1.28 mm, which is in accordance with the displacement of 1.6 mm obtained for the three 3D models tested.

Further studies should be conducted in order to test the dimensional stability performance of the 3D simulation model with two more cobblestones in the center and in the back area of the model. New techniques should also be tested so that Molloplast^®^ B could be attached without digital bone model destruction.

While 3D simulation models are important as a starting point in testing CDs’ performance, clinical in vivo studies are necessary to confirm those results. The use of mean calculations of the different measurements in each tooth mucosa, calculated by Dong Jian et al. [27], is also a limitation in this study because, in a real oral environment, the oral mucosa has different thicknesses in various areas of the high alveolar ridge.

## 5. Conclusions

SLA is a rapid prototyping system through which three-dimensional solid models are obtained. An in vitro simulation model with a reduced alveolar ridge with the same characteristics as mandibular edentulous patients was obtained using SLA. It offers an integrated solution, like the ability to be fixed to mechanical testing machines to conduct oral rehabilitation tests and the ability to simulate the oral mucosa’s performance, which have several advantages in the research area. The cobblestones’ design allowed easier adaptation to a transfer table, and the risk of errors in adapting it to a universal testing machine was reduced. Dimension stability tests confirmed the stability of the 3D simulation model, with more stability in the left and right areas. In short, simulation models of mandibular total edentulous patients obtained using AM, such as the one in this study, show sufficient precision for use in the research area.

## Figures and Tables

**Table 1:** Gingival thickness means calculated using the study by Dong Jian et al. [35].

Dental Position	Means		
	Buccal	Crest	Lingual
Central Incisors	1.7	2.2	1.9
Lateral Incisors	1.8	2.3	1.7
Cuspid	1.8	2.3	1.5
1° PM	1.7	2.1	1.4
2° PM	1.7	2.3	1.3
1° Molar	1.8	2.4	1.3
2° Molar	2.0	2.6	1.6

**Table 2:** RM ANOVA for left, right, and center attachments across sequence of tests.

Attachment	Test 1	Test 2	Test 3	F_(2, 4)_	*p*-Value	Partial η^2^
Center	0.0147 (0.0015)	0.0140 (0.0010)	0.0133 (0.0012)	F_(2, 4)_ = 16.00	0.057	0.89
Left	0.0063 (0.0015)	0.0060 (0.0010)	0.0067 (0.0006)	F_(2, 4)_ = 0.25	0.667	0.11
Right	0.0050 (0.0010)	0.0050 (0.0010)	0.0043 (0.0012)	F_(2, 4)_ = 4.00	0.184	0.67

The results are presented as the M (SD) of the three included models.

**Table 3:** Unilateral *t*-tests for different thresholds of stability difference for center, left, and right attachments.

N = 9	M (SD)	Dif = 0	Dif ≤ 0.005	Dif ≤ 0.010	Dif ≤ 0.015	Dif ≤ 0.020
Center	0.0140 (0.0012)	<0.001	<0.001	<0.001	0.980	>0.990
Left	0.0063 (0.001)	<0.001	0.002	>0.990	>0.990	>0.990
Right	0.0048 (0.00098)	<0.00	0.256	>0.990	>0.990	>0.990

The *t*-tests were calculated as negative unilateral *t*-tests for H0: μ ≤ difference threshold.

## Data Availability

The original contributions presented in this study are included in the article. Further inquiries can be directed to the corresponding author.
